# Evaluation of ventricular–vascular coupling with critical care metrics: An in silico approach

**DOI:** 10.14814/phy2.15920

**Published:** 2024-01-31

**Authors:** Lawrence J. Mulligan, Justin Ungerleider, Adam Friedman, Benjamin Sanders, Julian Thrash, Daniel Ewert, Ludmil Mitrev, Jeffrey C. Hill

**Affiliations:** ^1^ Department of Anesthesiology Cooper University Hospital Camden New Jersey USA; ^2^ Cooper Medical School of Rowan University Camden New Jersey USA; ^3^ North Dakota State University Fargo North Dakota USA; ^4^ University of North Dakota Grand Forks North Dakota USA; ^5^ School of Medical Imaging and Therapeutics Massachusetts College of Pharmacy and Health Sciences University Worcester Massachusetts USA

**Keywords:** aortic compliance, cardiac output, computational model, Eadyn, mechanical efficiency, pulse pressure, stroke work, ventricular–vascular coupling

## Abstract

Mean arterial pressure and cardiac output provide insufficient guidance for the management of intraoperative hypotension (IOH). In silico models offer additional insights into acute changes in hemodynamic parameters that may be encountered during IOH. A computational model (CM) generated parameters quantifying ventricular–vascular coupling, and pressure–volume construct across levels of aortic compliance (C_A_). We studied how a loss from normal‐to‐stiff C_A_ impacts critical care metrics of hemodynamics during vascular occlusion. Pulse pressure (PP), end‐systolic pressure (*P*_es_), arterial compliance (Art‐ca), arterial elastance (Art‐ea), and dynamic arterial elastance (Eadyn), along mechanical efficiency (ME) were measured at five levels of C_A_. A loss in C_A_ impacted all variables. During steady‐state conditions, PP, *P*_es_, and stroke work increased significantly as C_A_ decreased. Art‐ca decreased and Art‐ea increased similarly; Eadyn increased and ME decreased. During a decrease in preload across all C_A_ levels, arterial dynamics measures remained linear. The CM demonstrated that a loss in C_A_ impacts measures of arterial dynamics during steady‐state and transient conditions and the model demonstrates that critical care metrics are sensitive to changes in C_A_. While Art‐ca and Art‐ea were sensitive to changes in preload, Eadyn did not change.

## Graphical abstract

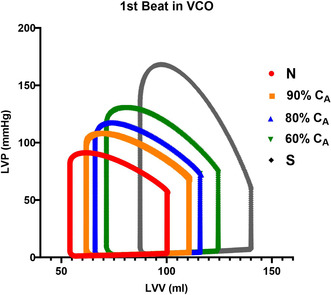

## 1 INTRODUCTION

Tools to improve intraoperative and postoperative care of patients following surgery have relied on mean arterial pressure (MAP), cardiac output (CO), systemic vascular resistance, and pulmonary capillary wedge pressure (Awad et al., 2022; Singh & Antognini, 2011) These parameters have been shown to include an informational lag regarding the patient's hemodynamic state but remained the foundation of patient care. Recent tools have been developed to improve the speed and focused insight on relevant parameters. Reliance on MAP and CO to monitor intraoperative hypotension (IOH) has led to the commercial development of new parameters to provide greater insight: Baroreceptors, carotid bodies, the cardio‐renal axis, and microvascular autoregulation control regulation of MAP and CO (Awad et al., 2022; Pinsky et al., 2022; Vos & Scheeren, 2019).

However, vascular aging and an accompanied loss in aortic compliance (C_A_) is not considered during management of these patients. A decrease in C_A_ and the resulting increase in arterial pulsatility leads to a loss of autoregulatory microvascular control, increasing the difficulty of controlling IOH (Georgianos et al., 2015; London et al., 2004; O'Rourke & Safar, 2005). Recent studies have investigated the use of Eadyn (pulse pressure [PP]/stroke volume [SV]), a dynamic version of arterial elastance (*E*_a_), to provide insight into IOH triage (Cecconi et al., 2015; Guarracino et al., 2013; Monge García et al., 2020).

Insight into this regulatory apparatus requires insight into how the cushion and conduit properties of the aorta impact a given patient's hemostatic level. With age, the aortic becomes less compliant, which may be coupled with a loss of the microvascular autoregulatory mechanisms and likely associated with a change in left ventricular function (Laurent et al., 2022; Laurent & Boutouyrie, 2021; Mitchell et al., 2004). The subsequent loss of cardiovascular homeostasis immediately following anesthesia induction or during the surgical case presents an additional burden to the variables listed above for the treating anesthesiologist. If the vascular tree has undergone early vascular aging (is stiffer than expected at a specific age), a loss in autoregulation will likely be more significant, and maintaining MAP within the guidelines may be more difficult.

The loss of C_A_ coupled with the difficulty of assessing and managing intraoperative blood pressure suggest that additional information would be helpful, and early identification and prompt management of hypotensive events can significantly impact patient outcomes (Laurent et al., 2022; Laurent & Boutouyrie, 2021). The American Society of Anesthesiologists set forth a standard rule that monitoring arterial blood pressure should not exceed an interval maximum of 5 min (Awad et al., 2022). Intraoperative blood pressure management calls for more advanced hemodynamic parameters to identify and predict patients who may experience hypotensive episodes and knowledge of C_A_ may improve the efficiency and use of the current monitoring paradigm.

The ventricular–vascular coupling (VVC) construct was first considered in the early work by Suga et al. (1973). The LV pressure–volume (PV) loop provided a load‐independent measure of the contractile state and time‐varying cardiac elastance (Kass & Kelly, 1992; Sagawa et al., 1977). During this study, the first cardiac cycle was used to evaluate *E*_a_ (*P*_es_/SV) using the upper left‐hand corner of the PV loop. Art‐ea is a poor metric for evaluating VVC (Chirinos et al., 2014; Ikonomidis et al., 2019). The recently proposed parameter, dynamic arterial elastance (Eadyn), which indexes PP and SV, has been utilized in the critical care setting to predict the volume status and provide an index of compliance responsiveness of the patient (Guarracino et al., 2013; Monge García et al., 2020; Persona et al., 2022). Higher values of Eadyn (>1) have been determined to reflect a cardiovascular system that adequately regulates blood pressure by efficiently increasing CO and arterial pressure when fluids are given (Awad et al., 2022; Guarracino et al., 2013; Monge García et al., 2020; Persona et al., 2022).

Further investigation is required to understand how Eadyn changes with manipulation of pressure and volume at different levels of C_A_, simulating the “real‐world” changes of loading conditions in the intensive care patient. Therefore, the aims of this study are (1) to evaluate how varying degrees of C_A_ impact beat‐to‐beat arterial compliance (Art‐ca) during steady state and transient conditions, (2) to evaluate how varying degrees of C_A_ impact effective Art‐ea, and Eadyn. To investigate these aims, a computational model (CM) previously developed was modified to include the addition of left ventricular PV with C_A_ values ranging from normal (baseline) to stiff (aged) and manipulation of preload via vena caval occlusions (VCOs).

## 2 METHODS

### 2.1 In silico computational model

A Simulink cardiopulmonary model was developed using MATLAB's MathWorks R2020b release (Natick, MA) based on previously validated work to simulate human cardiovascular experiments (Albanese et al., 2016; Cheng et al., 2016). The lumped parameter CM includes a five‐compartment systemic vascular system, pulmonary circulation, mechanics, and left and right ventricles and atria. The model is hemodynamically regulated through an autonomic neurological control system and local‐effect autoregulation through blood‐gas concentration.

### 2.2 Baseline model verification

The model was verified before the study by analyzing left ventricular PV loops and aortic pressures. Hemodynamic parameters of the MATLAB and Simulink model were tuned using the comparison to human physiologic cardiac PV responses seen in the work of Wiggers (Mitchell & Wang, 2014). Under normal compliance and resistance conditions, the model generated PV waveforms with systolic, diastolic, and PP of approximately 120 mmHg, 80 mmHg, and 40 mmHg, respectively.

The model developed by Ursino et al. includes five compartments that are part of the windkessel construct but with more granularity. Instead of one lumped windkessel, Ursino's models are constructed with many distributed lumped windkessels (Albanese et al., 2016; Cheng et al., 2016). The skeletal system, brain, heart/vascular tree, splanchnic bed, extra‐sphanchnic, and autonomic control of blood flow to and from these compartment mimic the body's many windkessels. In the initial windkessel that Westerhof used for his first manuscript, he included a capacitor and two resistors (Westerhof et al., 1971). Today, models have a capacitor (compliance), resistance (flow resistance), and inductor (inertance).

### 2.3 Experimental model setup

After verification, the model was adapted to fit the experimental setup and procedure of the canine experiment seen in Kelly et al. (1992). The model was modified to create the scenario used in invasive PV loop studies: right atrial pacing, autonomic nervous system (ANS) blockade, and a decrease in preload that mimics the VCO. The nominal vascular compliance and peripheral resistances were directly modified to simulate normal and stiff conditions that mimic human cardiac and arterial function and physiology (Albanese et al., 2016; Cheng et al., 2016). The model developed depicts the venous blood flow to the right atrium through the superior and inferior vena cava as the total summation of flow converging through the thoracic cavity veins. Flow convergence is modeled by pooling the total venous blood flow from the five vascular compartments.

### 2.4 Vena caval occlusion

**FIGURE 1:**
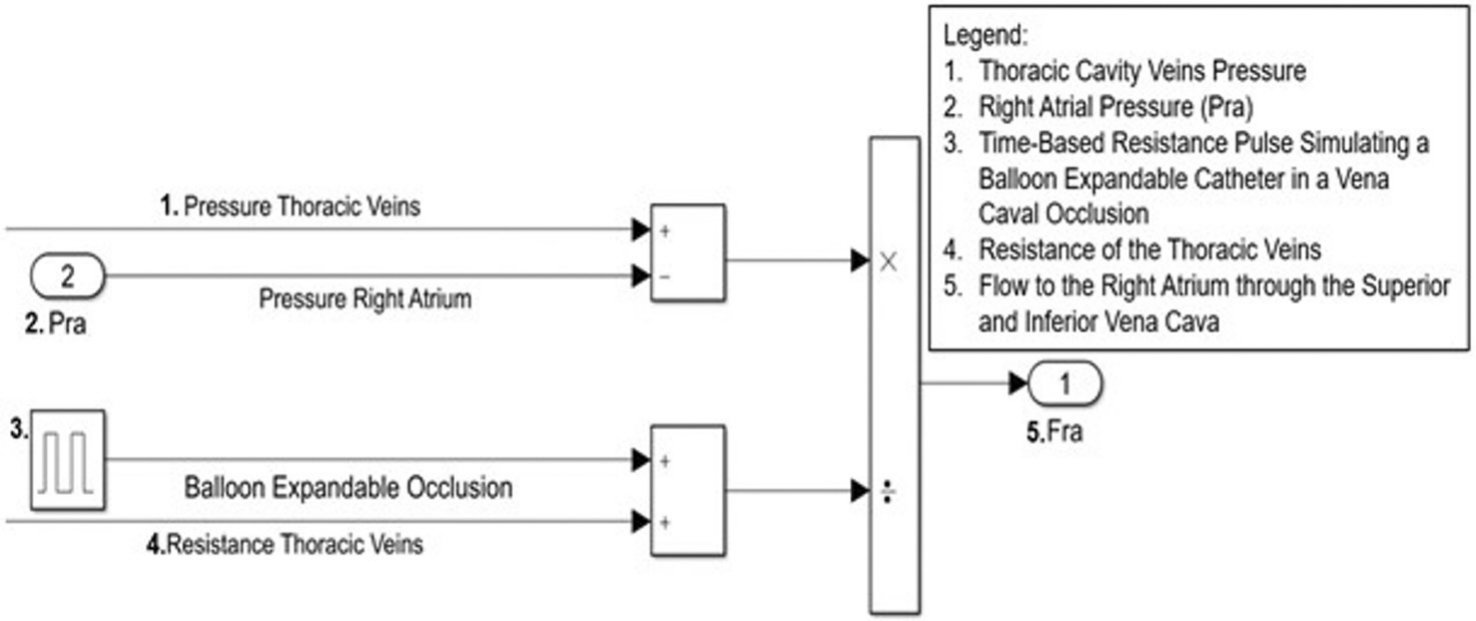
Simulink simulated balloon expandable vena caval occlusion (VCO) method. The autonomic blockade used routinely in acute and chronic canine studies to control heart rate during 10–12 s VCO was simulated by keeping sympathetic and parasympathetic reflex signals at a constant nominal value to prevent innervation on the cardiopulmonary system during the VCO period. F_ra_, blood flow to the right atrium; P_ra_, pressure gradient of the thoracic veins and right atrial pressure.

Occlusion of the venous return was simulated using a time‐based increase in the hemodynamic resistance of the thoracic cavity veins after allowing the system to reach a steady state 3 min before the occlusion (Figure 1). The occlusion occurred distal to the tricuspid valve and downstream of the five systemic vein sub‐compartments This in silico method is equivalent to occlusion of the vena cava via a balloon‐expandable catheter delivery system that replicates a reduced venous blood flow return.

Right atrial pacing was simulated at 80 bpm across the five levels of compliance during each VCO simulation. The normal and the stiff aorta's total CA and peripheral vascular resistance (R_T_) occurred by directly modifying the cardiopulmonary model systemic arterial parameters. Due to the large variance in vascular compliance and resistance between a human arterial system and a canine subject, the original canine normal and stiff Tygon arterial parameters were modified to synchronize within the hemodynamics of the cardiopulmonary model. The Art‐ca and resistances of the five compartments and aorta of the cardiopulmonary model were proportionally scaled to achieve a target equivalent compliance.

To simulate the canine arterial parameters of the Kelly et al. study in the human cardiopulmonary model, the total compliances and resistances for normal and stiff conditions were adjusted to accurately reflect physiologic human hemodynamics and were modified through proportional scaling of the arterial and aortic vascular compartments (Kelly et al., 1992). The normal canine total compliance was directly modified from a C_A_ = 1.65 mL/mmHg to C_A_ = 0.7 mL/mmHg while the stiff Tygon compliance of C_A_ = 0.19 mL/mmHg produced physiologically accurate hypertensive conditions within the human cardiopulmonary model. Peripheral vascular resistance was modified from the canine normal R_T_ = 3.04 mmHg.mL^−1^.s^−1^ to R_T_ = 1.28 mmHg.mL^−1^.s^−1^ while the resistance of the stiff Tygon conduit was kept at the Kelly et al. value of R_T_ = 3.66 mmHg.mL^−1^.s^−1^.

Beat‐per‐beat data was collected by simulating the VCO under all levels of compliance. The results were verified through an examination of total end diastolic volume (EDV), end‐systolic volume (ESV), end‐systolic pressure (*P*_es_), end‐diastolic pressure (EDP), PP, *d*P/*d*_tmax_, pressure–volume area (PVA), and the end‐systolic pressure–volume relationship (ESPVR). Validation of the model results was conducted by comparing the hemodynamic relationships seen in previous studies (Freeman, 1990; Kelly et al., 1992; Kolh et al., 2000). Data analysis was conducted using GraphPad Prism (Boston, MA).

After calculating the normal and stiff data, the Art‐ca and resistance were linearly scaled starting from the normal compliance (C_A_ = 0.7 mL/mmHg and R_T_ = 1.28 mmHg.mL^−1^. s^−1^) and reduced to the Tygon compliance to verify the linear behavior of the cardiopulmonary model. The normal compliance C_A_ was decreased by 10% to 0.63 mL/mmHg and resistance of R_T_ = 1.41 mmHg.mL^−1^. s^−1^; then by 20% to C_A_ = 0.56 mL/mmHg and R_T_ = 1.54 mmHg.mL^−1^.s^−1^; then by 40% to C_A_ = 0.42 mL/mmHg and R_T_ = 1.805 mmHg.mL^−1^.s^−1^; and finally to the stiff Tygon compliance and resistance value of C_A_ = 0.19 mL/mmHg and R_T_ = 3.66 mmHg.mL^−1^.s^−1^. The model parameters and generated output are shown in Table 1.

**TABLE 1:** Model parameters.

Model compliance (C_A_) (mL/mmHg)	Model resistance (R_T_)	Steady state arterial compliance	Steady state arterial elastance
	(mmHg. mL.s^−1^)	Art‐ca	Art‐ea
		(SV/*P*es)	(*P*es/SV)
Normal (0.7)	1.28	0.57	1.76
90% Normal (0.63)	1.41	0.50	1.99
80% Normal (0.56)	1.54	0.48	2.08
60% Normal (0.42)	1.805	0.47	2.13
Stiff (0.27)	3.66	0.39	2.54

Abbreviations: Art‐ca, arterial compliance; Art‐ea, arterial elastance; C_A_, aortic compliance; *P*es, end‐systolic pressure; SV, stroke volume.

### 2.5 Evaluation of cardiac function

Like human and pre‐clinical studies, LV volume was calculated for each beat during the simulated VCO. For the ESPVR, *P*_es_ and ESV were on was fit using a linear least‐square algorithm to the equation:

$$P_{\mathrm{es}}=\text{ESPVR}\left(E_{\mathrm{es}}-V_{o}\right)$$

where ESPVR is the slope of the relation and *V*_o_ is its volume‐axis intercept.

The PVA described by Suga et al. (1973) was calculated for each beat during the VCO. The efficiency of left ventricular energy transfer was evaluated using the method of Nozawa et al. (1988) using the equation:

$$\text{Mechanical efficiency}\;\left(\mathrm{ME}\right)=\left(\mathrm{SW}/\mathrm{PVA}\right)\times 100$$

### 2.6 Evaluation of arterial compliance/elastance

The CM provided five levels of static Art‐ca as described above. In addition, calculations of dynamic Art‐ca (SV/*P*_es_) and Art‐ea (*P*_es_/SV) were calculated. Eadyn (pulsatile “pulse pressure”/stroke volume [PP/SV]) was also calculated. The parameters were calculated during the first beat of the VCO (steady‐state) and the VCO process (transient). The CM parameters are shown in Table 1.

## 3 RESULTS

### 3.1 Impact of alterations in compliance on ventricular function

**FIGURE 2:**
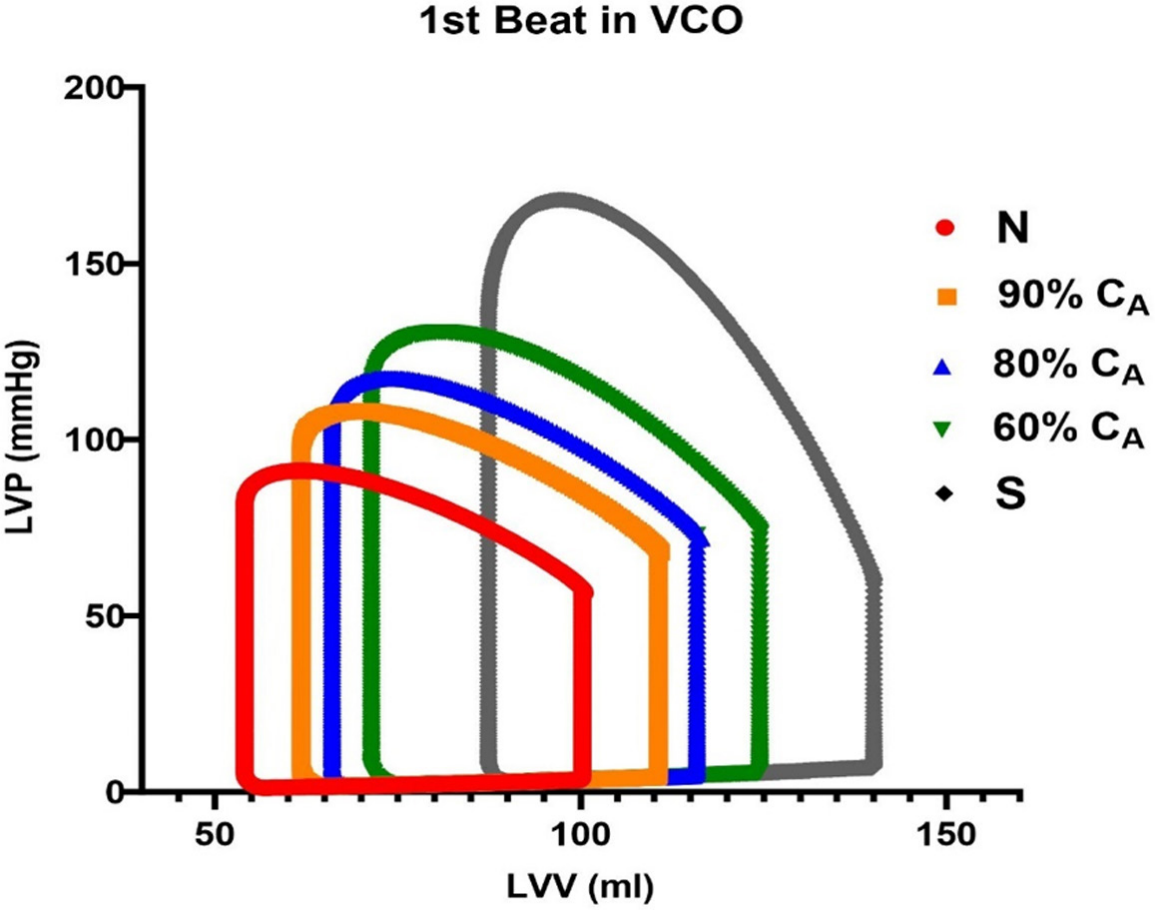
The impact of a loss in aortic compliance on steady state cardiac function. The first cardiac cycle from the vena caval occlusion (VCO) maneuver for each compliance is shown with each loop moving to the right. LVP, left ventricular pressure; LVV, left ventricular volume.

**FIGURE 3:**
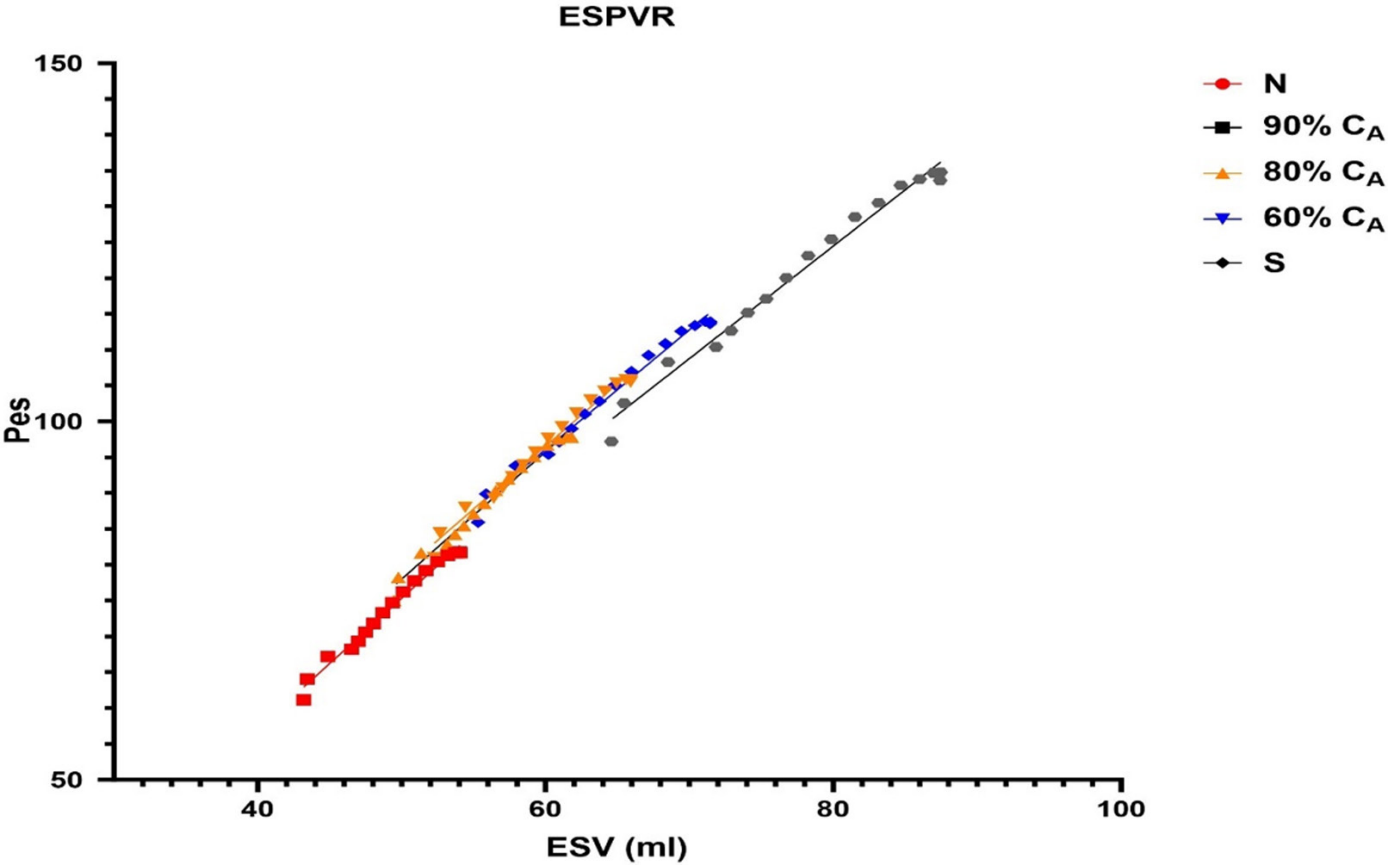
The impact of a loss in aortic compliance on end‐systolic pressure–volume relationship (ESPVR). The data were collected during a vena caval occlusion (VCO) maneuver for each compliance. The slopes are similar but there was a small reduction in ESPVR at the stiff aortic compliance (C_A_) (see Section 4).

The changes in C_A_ on ventricular function variables are shown in Tables 2 and 3. The following hemodynamic changes occurred as C_A_ decreased from normal to stiff. The PV plane moved to the right (Figures 1 and 2), and the LV end‐diastolic volumes (LVEDV) increased from 100.5 to 140.1 mL at the stiff C_A_. LV end‐systolic volumes (LVESV) increased from 54.1 to 87.4 during the stiff C_A_, and SV increased from 46.5 to 52.7 mL (Table 2). Associated with the change in C_A_, LV *P*_es_ increased from 81.7 mmHg at the normal setting to 133.8 mmHg during the stiff CA. During the loss in C_A_, LVEDP increased from 5.0 to 8.4 mmHg. Along with an increase in *P*_es_ and LVEDP, PP increased from 33.4 to 106.6 mmHg, and the changes in stroke work (SW) were also significant (3830 to 7313 mmHg mL). Measurement of left ventricular contractility with ESPVR decreased from 1.80 to 1.44 (Figure 3) during the loss in C_A_. As C_A_ decreased, an interesting contrast occurred with the ME variable, as function of ESV. As ESV increased during the first cardiac cycle of the respective VCO, a small decrease in ME of 2% was observed. This continued to the 60% C_A_ setting. This was due to the increased in stroke‐work, related to the increase in *P*_es_. Across the five C_A_'s, the change in ME during the VCO was eliminated at the Stiff setting (Table 4), beginning at 61% and ending at 60%.

**TABLE 2:** Impact of change in aortic compliance on LV function.

Aortic compliance	EDV	ESV	*P*_es_	EDP	SW	ESPVR	Mech eff
	(mL)	(mL)	(mmHg)	(mmHg)	mmHg.mL	(mmHg.mL)	(%)
Normal	100.5	54.1	81.7	5.0	3830	1.80	72.2
90%	110.9	61.8	97.8	5.9	4802	1.76	68.6
80%	116.7	65.9	105.5	6.3	5359	1.73	67.4
60%	124.7	71.4	113.7	7.0	6150	1.65	66.4
Stiff	140.1	87.4	133.6	8.4	7313	1.44	60.8

Abbreviations: EDP, end‐diastolic pressure; EDV, end diastolic volume; ESPVR, end‐systolic pressure–volume relationship; *P*_es_, end‐systolic pressure; ESV, end‐systolic volume; SW, stroke work.

**TABLE 3:** Impact of change in aortic compliance on ventricular–vascular coupling.

Windkessel parameters			Critical care metrics		
C_A_	SV	PP	Art‐ca	Art‐ea	Eadyn
Normal	46.5	33.4	0.57	1.76	0.72
90%	49.1	38.8	0.50	1.99	0.79
80%	50.8	44.0	0.48	2.08	0.87
60%	53.3	56.0	0.47	2.13	1.05
Stiff	52.7	106.6	0.39	2.53	2.02

Abbreviations: Art‐ca, arterial compliance; Art‐ea, arterial elastance; C_A_, aortic compliance; Eadyn, dynamic arterial elastance; PP, pulse pressure; SV, stroke volume.

**TABLE 4:** Impact of change in aortic compliance on mechanical efficiency during a vena caval occlusion.

	Normal	90%	80%	60%	Stiff
First cardiac cycle‐ME	72%	69%	67%	67%	61%
Last cardiac cycle‐ME	61%	59%	59%	59%	60%

### 3.2 Impact of alterations in compliance on metrics of ventricular–vascular coupling

**FIGURE 4:**
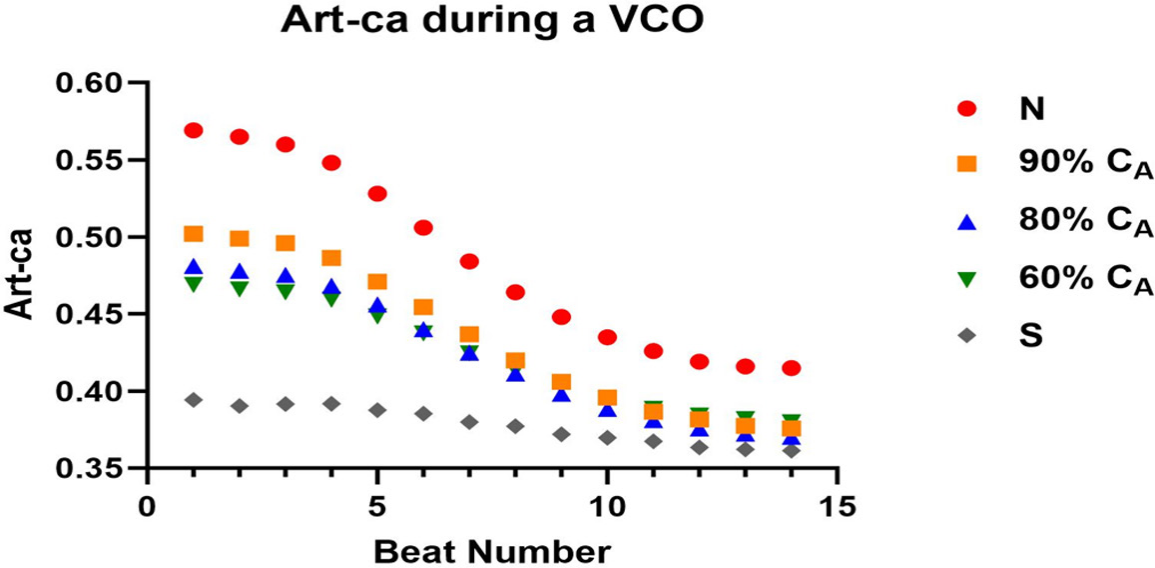
The impact of a loss in aortic compliance (C_A_) on the transient arterial compliance (Art‐ca) during a vena caval occlusion (VCO).

**FIGURE 5:**
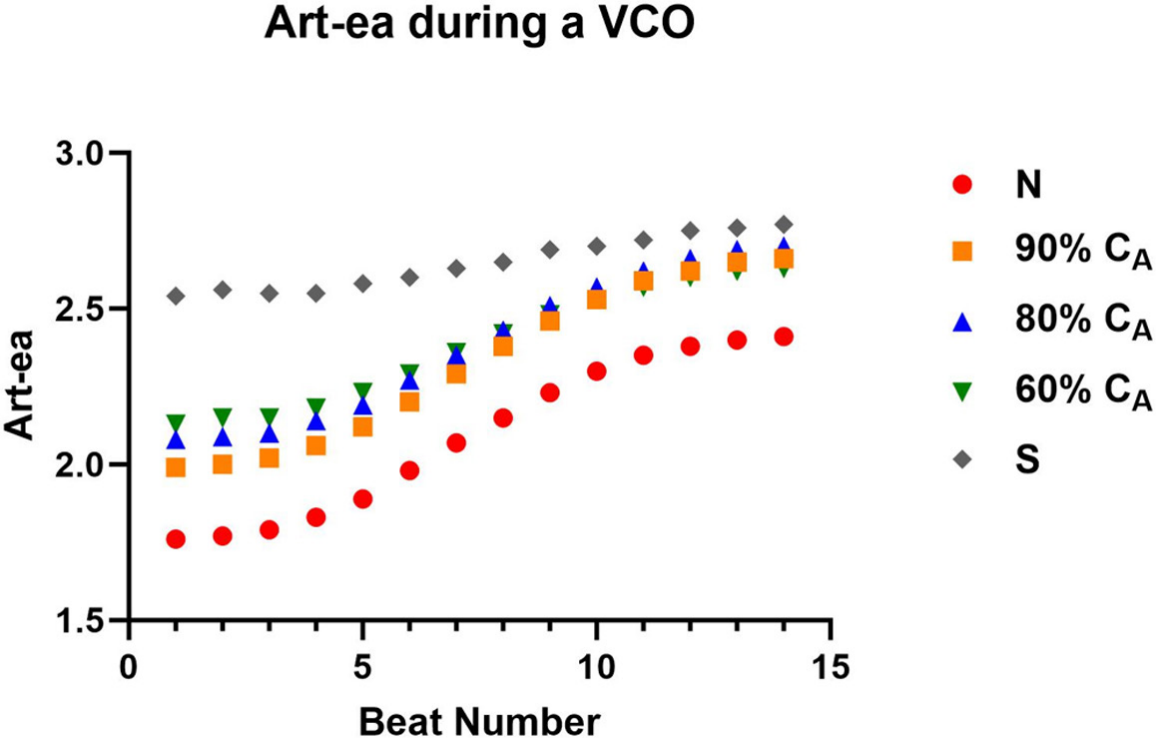
The impact of a loss in aortic compliance (C_A_) on transient effective arterial elastance (Art‐ea) during a vena caval occlusion (VCO).

**FIGURE 6:**
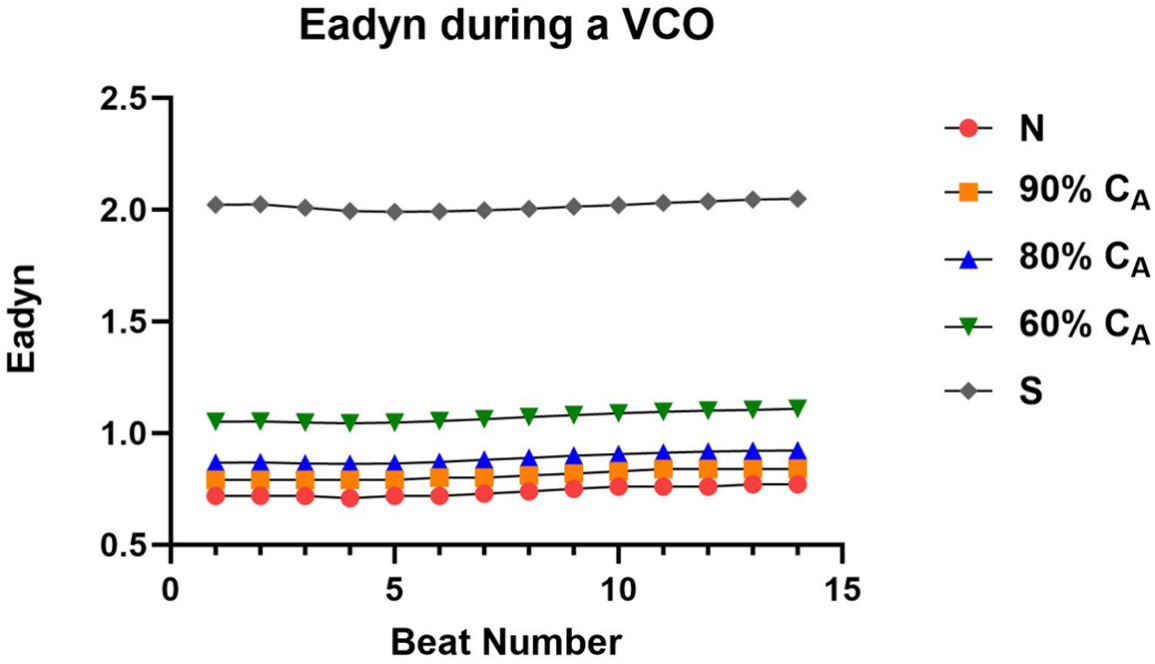
The impact of a loss in aortic compliance (C_A_) on the transient dynamic arterial elastance (Eadyn) during a vena caval occlusion (VCO).

**FIGURE 7:**
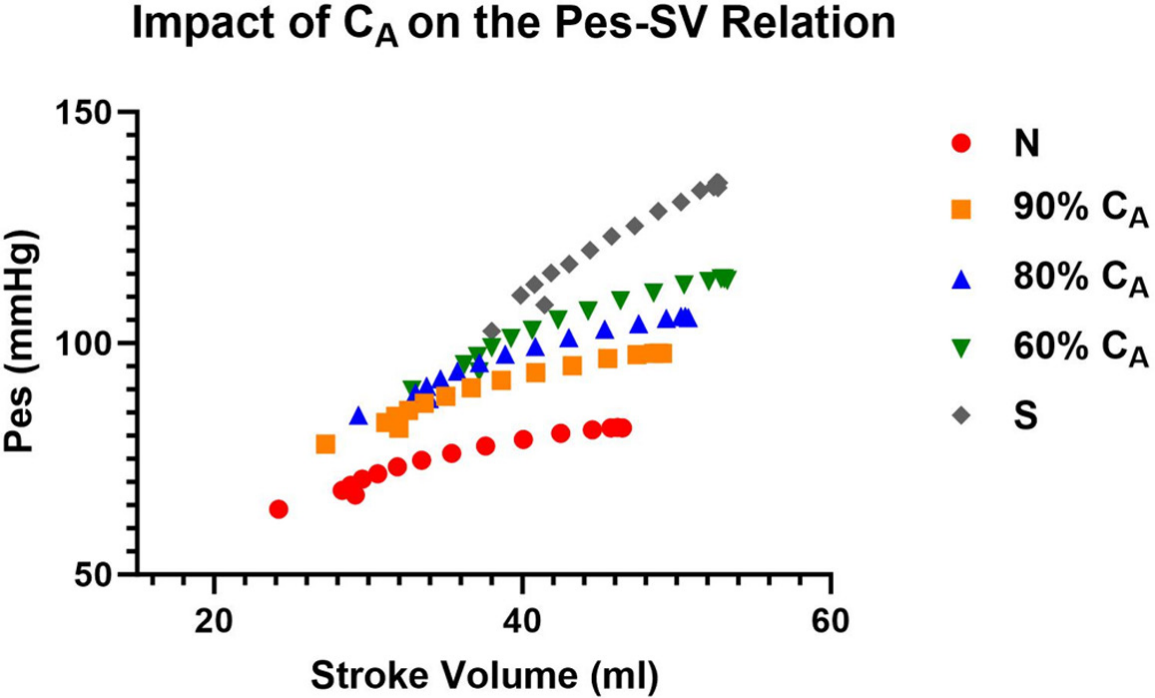
The impact of a loss in aortic compliance (C_A_) on the end‐systolic pressure (*P*_es_)–stroke volume relation during a vena caval occlusion (VCO).

**FIGURE 8:**
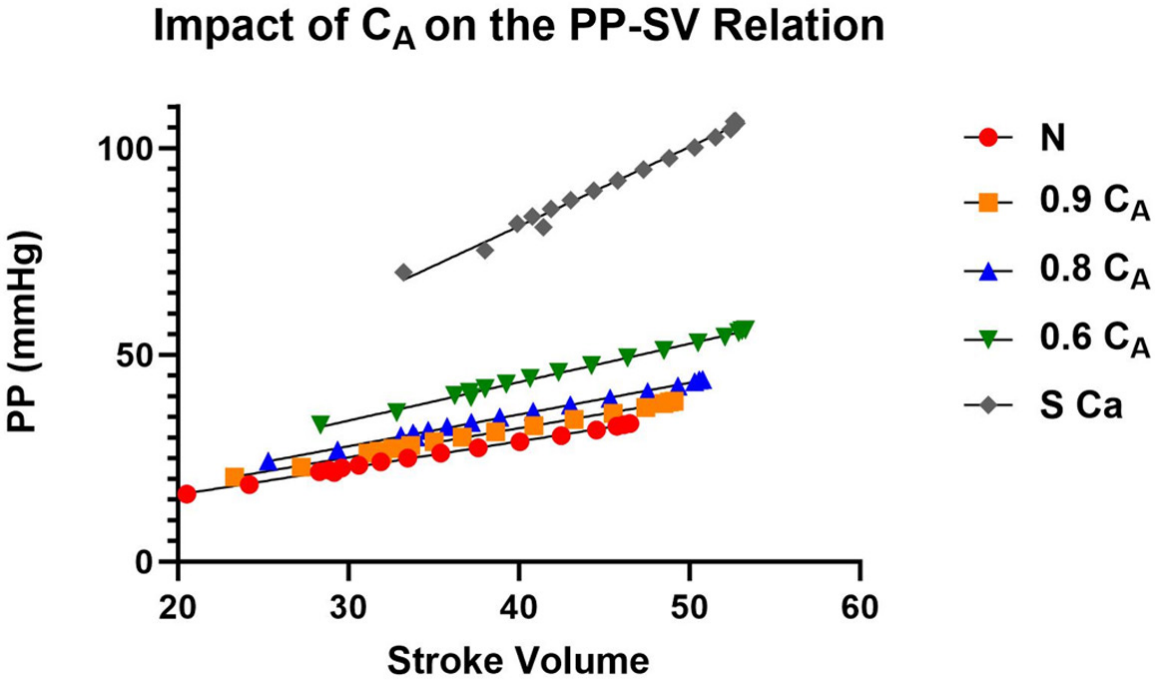
The impact of a loss in aortic compliance (C_A_) on the pulse pressure (PP)–stroke volume (SV) relation during a vena caval occlusion (VCO).

**FIGURE 9:**
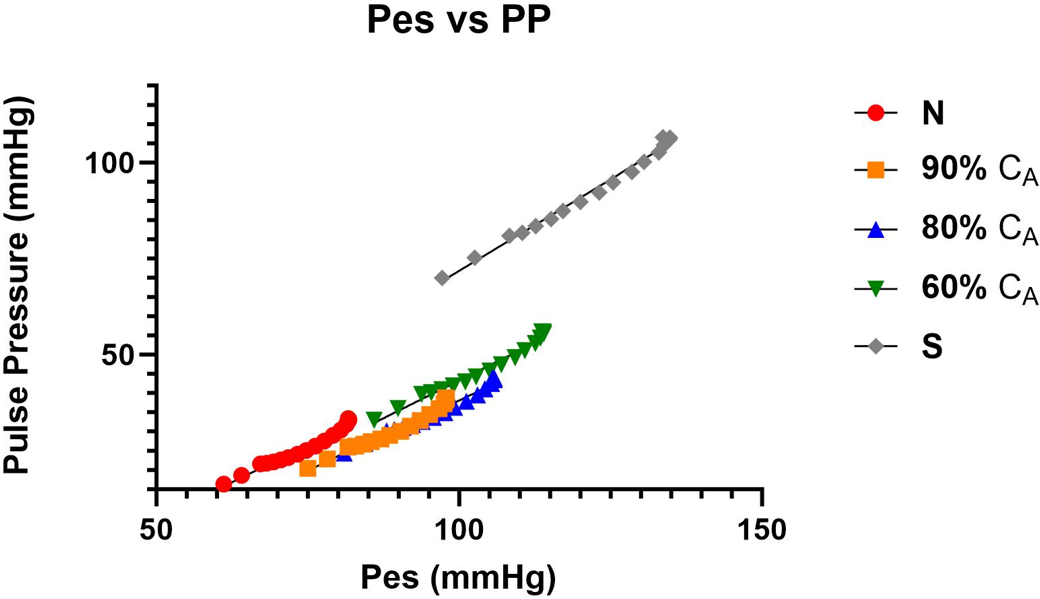
Relationship between end‐systolic pressure (ESP) and pulse pressure (PP) during vena caval occlusions (VCOs) at the five levels of aortic compliance (C_A_).

As expected, the loss in C_A_ led to similar and opposite changes in steady‐state Art‐ca and Art‐ea (Tables 1 and 3). During the VCO maneuver, intended to mimic changes in loading condition, the change in beat‐to‐beat Art‐ca and Art‐ea was preload dependent, and the level of dependence decreased as C_A_ decreased (Figures 4 and 5). However, the impact of the preload maneuver did not have the same effects on Eadyn (Figure 6). Eadyn uses PP, and Art‐ca and Art‐ea use *P*_es_. We evaluated the relationship between stroke volume and the two pressures at each C_A_ during the VCO (Figures 7 and 8). Eadyn remained linear but was not preload‐dependent. The relationship between PP and *P*_es_ was not linear (Figure 9) at any level C_A_. The impact of a loss in C_A_ on ME provided new observations. From the normal setting of C_A_ to the stiff setting, a loss in ME of 20% was observed (72%–61%) at steady state. During the VCO, the impact of a loss in C_A_ on ME increased. By the stiff setting, there was no change in ME, which was in contrast to the other settings (Table 4).

## 4 DISCUSSION

The in silico CM was modified to evaluate four questions: (1) Does a loss in C_A_ impact the left ventricular PV‐derived variables? (2) Does a loss in C_A_ impact variables representing VVC? (3) Does the does the loss in (C_A_) impact the clinically focused critical care metric, Eadyn along with Art‐ca? And is (4) Eadyn responsive to changes in preload? To accomplish this, we used a lumped parameter approach. The model was modified to provide insights from the left ventricular PV plane during alterations in preload and afterload. The four objectives were achieved, and the PV loop data generated by the model are in agreement with previous in vivo studies. These findings provide a critical appraisal of tools used in the critical care setting.

The model developed by Ursino et al. includes five compartments that are part of the windkessel construct but with more granularity. Instead of one lumped windkessel, Ursino's models are constructed with a distributed lumped windkessel approach (Albanese et al., 2016; Cheng et al., 2016; Little et al., 1989). The skeletal system, brain, heart/vascular tree, splanchnic bed, extra‐sphanchnic and autonomic control of blood flow to and from these compartments mimic the circulation's windkessels. In the initial windkessel work by Westerhof, the model included a capacitor and two resistors. Today, models have a capacitor (compliance), resistance (flow resistance) and inductor (inertance).

### 4.1 Impact of vascular aging on cardiac function

The CM produced data that suggest the following: first the model produced PV loops measured during the first beat in a VCO that displayed the expected rightward shift along the left ventricular volume axis as C_A_ decreased, second, the loss in C_A_ resulted in a graded response, providing insight into how the loss of C_A_ may impact cardiac function and finally, the existing metrics to evaluate response to preload maneuvers and subsequent changes in PP responsed to changes in preload while Eadyn did not. The steady‐state loss in C_A_ also led to increases in LVESV, LVEDP, PP, SV, and SW. Along with the changes in volumes and left ventricular pressures during the loss in C_A_, ESPVR decreased slightly from 1.80 to 1.44, along with a steady loss in ME.

### 4.2 Impact of vascular on VVC

The CM allowed for an investigation of VVC across a wide range of vascular compliances. As Eadyn has been linked to changes in arterial stiffness or compliance, we investigated the impact of simulated vascular aging on cardiac and vascular function. From the PV construct, we determined that the CM behaved in a manner similar to pre‐clinical and clinical studies regarding cardiac function, preload, ME and contractility (Chen et al., 1998; Freeman, 1990; Kolh et al., 2000). Using the CM, we evaluated how Art‐Ca, Art‐ea and Eadyn responded at five levels of C_A_. The steady‐state data suggest that Art‐ca and Art‐ea parameters behave as expected as C_A_ decreases in vascular aging (Table 3). Eadyn also changes during steady‐state conditions, as C_A_ decreased Eadyn increased from 0.72 to 2.02.

During the VCO, Art‐ca and Art‐ea behaved in an inverse manner (Figures 4 and 5). The Eadyn construct suggests that VVC is good or optimal above a value of 1.0. The CM showed that a normal C_A_ was associated with a value of 0.72 and only when the CM simulated a stiff vascular bed, did Eadyn rise above 1.0. The normal C_A_ for ESPVR and ME are aligned with human values, while the Eadyn value of 0.72 is 50% of that measured in patients with an average ejection fraction of 54% (Kameyama et al., 1992).

Our observation regarding Eadyn may be a function of the model. The other parameters from the PV construct are aligned with previous studies and the behavior of Art‐ea is also aligned. During the VCO, new observations include a dynamic response of Art‐ca and Art‐ea (Figures 4 and 5) that mirror each other and a lack of a response in Eadyn (Figure 6). The cause of this difference is likely the use of PP in the Eadyn tool versus *P*_es_ (Figures 7 and 8) where stroke volume on the x‐axis is the common element. The slopes and intercepts appear to be quite different between PP and *P*_es_. In the Monge‐Garcia dataset, Art‐ea is 1.29 at baseline and 0.80 following phenylephrine with a reverse during sodium nitroprusside (1.13–2.0). These data are from animal models, while the CM mimics human data, but the Eadyn findings during the phenylephrine and sodium nitroprusside decreased from 1.51 to 0.98 and increased from 1.36 to 1.79. This suggests that during phenylephrine, PP decreased more than SV while during nitroprusside SV increased more than PP.

The Eadyn parameter was intended to provide insight using critical care parameters to guide decision‐making in patients who may require volume. As C_A_ decreases with age, the PP increases, and the long‐term increase in afterload leads to a loss in SV. In the CM, left ventricular function remains normal, enabling the study to address vascular aging only. This resulted in a significant increase in PP and a larger Eadyn than would be seen in a critical care patient. By modifying the model proposed by Albanese et al. (2016), we were able to investigate how the loss in C_A_ impacted Eadyn. The Eadyn variable was based on the effective arterial elastance (*E*_a_ or Art‐ea) construct. In the PV construct, *E*_a_ is only considered for the first cardiac cycle of the PV loop data, and this is due to *E*_a_ changing during the VCO and, therefore, cannot represent the Art‐ea similar to ventricular elastance. However, Eadyn was intended to evaluate pulsatile changes in SV and PP.

The premise of Eadyn was to determine if a patient would be responsive to fluids. The addition of fluids would be expected to change preload, MAP, and stroke volume in a homeostatic circulation. The VCO maneuver has been shown to drop preload and afterload without changing sympathetic control of the circulation. The CM VCO provided a close similarity to this technique.

In contrast to the relationship between Eadyn and Art‐ca, we investigated how the loss of C_A_ impacted the beat‐to‐beat change in both Art‐ea and Eadyn during the VCO, respectively (Figures 5 and 6). During a VCO, the characterization ESPVR is linear, providing the load‐independent measure of contractile function. However, the changes in the vascular side (*E*_a_) are not linear. In Figures 4 and 5, the impact of a loss in C_A_ is clear, and the impact of the loss on Art‐ca and Art‐es during the VCO, as the vascular tree stiffens, is clear. The consequences of this behavior are two‐fold. First, the need to move away from effective arterial elastance (*E*_a_, Art‐ea) as part of the VVC paradigm is clear and recent work provides the basis for this (Chirinos et al., 2014). *E*_a_ is used only during steady state conditions and depends on heart rate and peripheral resistance as is C_A_. Second, the change in beat‐to‐beat Art‐ca during a VCO is evident at the five levels of C_A_. This evidence supports the load dependence of assessing VVC across the spectrum of vascular aging.

### 4.3 Comparison of arterial elastance/compliance with Eadyn

The transition of the use of *E*_a_ to Eadyn includes the use of PP compared with *P*_es_. Physiologically, the use of PP adds to the complexity as PP is known to increase with vascular aging (Chen et al., 1998; Chirinos, 2022; Climie et al., 2023; London et al., 2004). In addition, the relationship between change the in PP and stroke volume during the process of vascular aging is complicated. As the Eadyn metric calculates stroke volume from the radial arterial pressure line, it is reasonable to conclude that in the patient population over 50 years, which was simulated to a degree at the 60% and stiff settings, an error may develop in the metric.

We were interested in how the change in C_A_ impacted Art‐ca. In Figure 4, a loss in C_A_ from normal to stiff led to a lower value and the contrast of the beat‐to‐beat decline demonstrates the load dependence of the variable. Evaluation of Eadyn showed a lack of change as C_A_ decreased, suggesting the lack of sensitivity of parameter. The relationship between *P*_es_ and stoke volume was curvilinear remained linear (Figure 7) but the relationship between PP and stroke volume was linear. Also, the impact of a loss in C_A_ led to gradual separation in the relationship while the loss in C_A_ created similar slopes but greater separation with PP (Figure 8).

### 4.4 Model limitations

The major limitation of the study is the use of a CM to stimulate the human cardiovascular system compared to the use of human subjects. Due to the invasive and specific nature of the parameters being measured (Art‐ca, ESPVR, SV, and ME) and the use of a change in loading conditions across degrees of vascular aging, utilizing human subjects is not possible. The initial focus of the model was to simulate vascular aging with a normal heart. The next step could focus on changing the function of the heart in a similar manner both with and without disease. Invasive studies in a large sample of patients aged 25–75 years to generate the VVC and Art‐ca data who are undergoing cardiac catheterization or cardiac surgery is not realistic. Other have used in vivo and in silico methods to evaluate similar questions (Moulton & Secomb, 2023; Pagoulatou et al., 2021; Pagoulatou & Stergiopulos, 2017). Modeling these hemodynamic situations at varying C_A_s and preloads served as a reasonable and effective way to assess Eadyn. Extrapolating these data and the associated relationship in human subjects is an opportunity to improve the validity and better understand the potential clinical utility. A possible next step would be to use human models with varying degrees of aortic stiffness and volume status to assess Eadyn, and ultimately the relationship to predicting fluid responsiveness. As Eadyn continues to become more accessible, less invasive and a standard part of anesthesia practice, future studies can involve human subjects to better relate this information to the human cardiovascular system as opposed to simulation via CMs.

## 5 CONCLUSION

The development of noninvasive or minimally invasive tools for assessing fluid responsiveness in surgical patients continues to build on fundamental hemodynamics. The creation of Eadyn as tool to assist with this goal appears to provide important guidance regarding patient volume status. However, the degree of vascular aging, minimal to severe, that may have developed in the patient, can impact the responsiveness of this variable, as the computational model has demonstrated.

## FUNDING INFORMATION

The authors declare that this report did not receive any specific grant from public, commercial, or not‐for‐profit funding agencies.

## ETHICAL APPROVAL

The authors declare that the work described has been carried out in accordance with The Code of Ethics of the World Medical Association (Declaration of Helsinki) for experiments involving humans. There were no human subjects included in this study.

## Data Availability

Julian Thrash can be reached out at julian.thrash@ndsu.edu for further information on the CM model and code used to modify the CM. Questions regarding the datasets can be directed to Larry Mulligan at mulligan-lawrence@cooperhealth.edu.
